# Refractive Index Sensor Based on a Metal-Insulator-Metal Bus Waveguide Coupled with a U-Shaped Ring Resonator

**DOI:** 10.3390/mi13050750

**Published:** 2022-05-09

**Authors:** Xiaoyu Zhang, Shubin Yan, Jilai Liu, Yifeng Ren, Yi Zhang, Lifang Shen

**Affiliations:** 1 School of Electrical Engineering, Zhejiang University of Water Resources and Electric Power, Hangzhou 310018, China; zhangxiaoyu9725@163.com (X.Z.); liujl@zjut.edu.cn (J.L.); zhangyi@zjweu.edu.cn (Y.Z.); shenlf@zjweu.edu.cn (L.S.); 2 Joint Laboratory of Intelligent Equipment and System for Water Conservancy and Hydropower Safety Monitoring of Zhejiang Province and Belarus, Hangzhou 310018, China; 3 School of Electrical and Control Engineering, North University of China, Taiyuan 030051, China; renyifeng126@126.com

**Keywords:** Fano resonance, SPPs, U-shaped ring resonator, sensor, metal-insulator-metal

## Abstract

In this study, a novel refractive index sensor structure was designed consisting of a metal-insulator-metal (MIM) waveguide with two rectangular baffles and a U-Shaped Ring Resonator (USRR). The finite element method was used to theoretically investigate the sensor’s transmission characteristics. The simulation results show that Fano resonance is a sharp asymmetric resonance generated by the interaction between the discrete narrow-band mode and the successive wide-band mode. Next, the formation of broadband and narrowband is further studied, and finally the key factors affecting the performance of the sensor are obtained. The best sensitivity of this refractive-index sensor is 2020 nm/RIU and the figure of merit (FOM) is 53.16. The presented sensor has the potential to be useful in nanophotonic sensing applications.

## 1. Introduction

Surface plasmon polaritons (SPPs) are electromagnetic surface waves generated by the interaction between an external light field and free electrons in metal, which can reach the maximum field intensity on the surface and decay exponentially along the direction perpendicular to the interface [1,2]. SPPs can be excited in two ways, by electrons or light waves. When the size of a specific nanostructure is reached, SPPs can break through the limited conventional diffraction and control light on the nanoscale [3,4]. SPPs have three characteristics: low dimension and high intensity and subwavelength, which make them a good energy and information carrier, and their ability to combine subwavelengths can be used to make various optical devices [5], such as wavelength demultiplexers [6,7], plasmonic filters [8,9], logic gates [10], couplers [11], and sensors [12,13]. 

In recent years, refractive index sensors based on a metal-insulator-metal (MIM) waveguide have attracted wide attention because of their strong lateral confinement, easy manufacturability, low propagation loss, and shorter transmission length [14,15]. Sensors based on MIM waveguides can also produce nonlinear optical effects such as electromagnetically induced transparency (EIT) and Fano resonance. Fano resonance is a kind of scattering resonance phenomenon that can produce an asymmetrical line shape. The shape of Fano resonance is caused by interference in the scattering amplitude of the discrete narrow-band mode and successive wide-band mode. Due to the rapidly changing amplitude and phase, Fano resonance has a narrower full width at half maximum (FWHM) [16]. Because of the unique linear shape of Fano resonance, the smaller FWHM has the higher electromagnetic field binding ability, which is widely used to characterize the resolution of the instrument.

In addition, Fano resonance is very sensitive to structural parameters and refractive index changes, which is very helpful for the preparation and improvement of refractive index sensors [17]. It is an important method for designing refractive index sensors by using a waveguide coupling resonator to generate Fano resonance. Zhou et al. [18] proposed an MIM waveguide consisting of two baffling resonators and a ring resonator; this structure can increase the sensitivity to 1650 nm/RIU. Zhang et al. [19] put forward an MIM waveguide which can support Fano resonance with a sensitivity of 1268 nm/RIU. Liu et al. [20] designed a refractive-index nanosensor, which can reach 1510 nm/RIU sensitivities. Zhang et al. [21] proposed a tooth cavity-coupled ring-splitting cavity structure obtaining a sensitivity of 1200 nm/RIU. Herein, the proposed nanosensor, with a simple structure, can achieve a high sensitivity of 2020 nm/RIU with a FOM of 53.16. 

In this article, a novel refractive index sensor structure consisting of a USRR and an MIM waveguide with two rectangular baffles is propounded theoretically. The USRR cavity can be represented with two parameters, which greatly reduces the difficulty of fabrication. Compared with other cavity structures, a USRR is more sensitive to the change in structural parameters, which is helpful for researching high sensitivity refractive index sensors. The standardized $H_{z}$ field distributions and the propagation characteristics were theoretically proposed by the finite element method (FEM).

## 2. Materials and Methods

**Figure 1:**
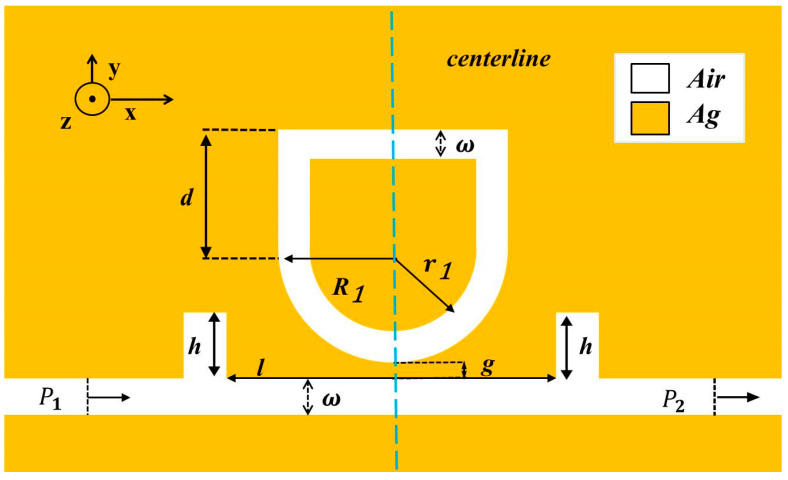
2D schematic diagram of a USRR resonant cavity coupled with an MIM waveguide with two rectangular baffles.

A schematic diagram of the designed structure is shown in Figure 1. A U-shaped cavity was chosen because it combines the advantages of circular and rectangular resonators. The rectangular resonator can change the vertical distance independently of the horizontal direction, and the straight waveguide is very beneficial to the transmission of light, but the coupling of the straight waveguide and MIM waveguide will cause a larger FWHM, and the perception performance will decrease. A circular resonator is the best choice, and displays a good performance in coupling with MIM waveguide. However, if the radius is increased, the increased cavity area in the vertical direction will lead to a decline in the sensor’s performance and an increased loss, thus affecting the spread performance. In the end, we chose the USRR structure. The coupling mode between the resonator and bus waveguide can be divided into side coupling and shoulder coupling. The structure proposed in this work adopts the side coupling mode. The geometric parameters of the structure settings are as follows: $R_{1}$ and $r_{1}$ are the outer radius and inner radius of the USRR taken separately; the height of the USRR is described as d; g is the coupling gap between the USRR resonant cavity and the bus waveguide; the distance between the two rectangular baffles and the height of them are denoted as l and h. The input and output ports are $P_{1}$ and $P_{2}$, respectively, and ω represents the width of the MIM waveguide. ω is usually set to 50 nm to ensure only T$M_{0}$ mode exists in the waveguide [22]. 

The TM mode equation is [23,24]:

(1) $$\tan h\left(k\omega \right)=-2{\mathrm{kp}\alpha }_{c}/\left(k^{2}+p^{2}k^{2}\right)$$

where p = $\varepsilon_{\mathrm{in}}/\varepsilon_{m}$, k is the wave vector, and $\alpha_{c}$ = ${\left[{k_{0}}^{2}\times \left(\varepsilon_{\mathrm{in}}-\varepsilon_{m}\right)+k\right]}^{1/2},\;\varepsilon_{m}$ and $\varepsilon_{\mathrm{in}}$ represent the permittivity of metal and dielectric, respectively. In free space, k can be depicted as $k_{0}$ = $2\pi /\lambda_{0}$.

A 2D diagram can be used instead of a 3D diagram for simple calculation. The yellow and white parts were filled with silver and air, respectively. A sufficiently thick silver layer can be prepared on a silicon substrate by chemical vapor deposition (CVD). The desired structure can be obtained by electron beam etching on the silver layer. The silver was chosen as the filling metal, which is due to its higher electric field and lower power consumption. The relative permittivity of air is 1, and the relative permittivity of silver can be acquired using the Debye–Drude dispersion model:

(2) $$\varepsilon_{\mathrm{Ag}}\left(\omega \right)=\varepsilon_{1}\left(\omega \right)+\varepsilon_{2}\left(\omega \right)i=1-\frac{{\omega_{p}}^{2}\tau^{2}}{1+\omega^{2}\tau^{2}}+\left(1-\frac{\omega_{p}{\omega_{p}}^{2}\tau }{\omega \left(1+\omega^{2}\tau^{2}\right)}\right)i\;$$

where ω represents the angular frequency of the light, $\omega_{p}\;\left(\omega_{p}=1.38\times 10^{16}\right)$ can be expressed as the plasma frequency of silver, and $\tau \left(\tau =7.35\times 10^{15}\right)$ is the relaxation time [25,26]. 

The performances of the proposed structure can be evaluated by two parameters, sensitivity (S) and figure of merit (FOM), which can be reached as follows [27]:

(3) S=Δλ/Δn

(4) FOM=S/FWHM 

where Δλ and Δn represent the change in resonance wavelength and refractive indices, respectively.

Before the sensor was manufactured, the transmission spectrum of the structure was numerically simulated with COMSOL Multiphysics 5.4. Boundary conditions can be divided into absorptive boundary conditions and periodic boundary conditions. The absorbing boundary condition was established by a perfect matching layer to absorb spilled waves. By setting a high loss layer perfectly matching the dielectric impedance of the adjacent region in the boundary region, the electromagnetic wave decays rapidly without reflection until it is completely absorbed. The superfine mesh was selected to improve segmentation accuracy and guarantee perfect segmentation.

## 3. Results

**Figure 2:**
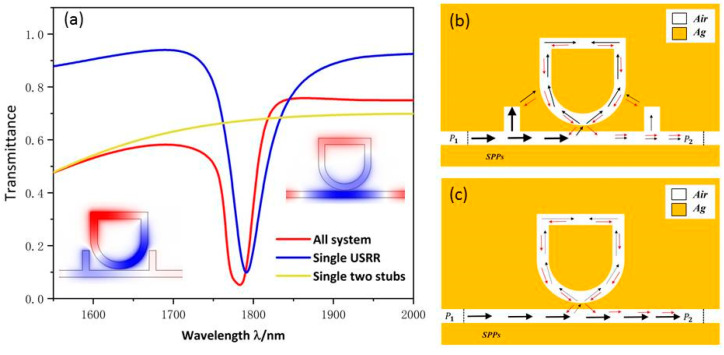
(**a**) Transmittance spectra of the single two baffles (yellow line), single USRR resonant cavity (blue line), and all system (red line); (**b**) The SPP pathway schematic of the system; (**c**) The SPP pathway schematic of the single USRR structure.

Since Fano resonance plays a significant role in the sensitivity of the sensor, we will investigate the formation and characteristics of Fano resonance. The structural parameters are as follows: $R_{1}=210\;\mathrm{nm},{\;r}_{1}=R_{1}-50,\;d=205\;\mathrm{nm},\;h=145\;\mathrm{nm},\;l=440\;\mathrm{nm},\;g=10\;\mathrm{nm}$. The method of Fano resonance excitation is divided into waveguide side coupled cavity excitation and symmetry breaking excitation. In this work, we used the waveguide side coupled resonator excitation mode. Fano resonance is caused by the interaction of the successive wide-band mode, which is excited by the bus waveguide with two rectangular baffles and the discrete narrow-band mode formed by the USRR resonant cavity. To evaluate the different transmission characteristics, three structures, with the whole system (red line), the single USRR resonant cavity (blue line), and two symmetrical rectangular baffles (yellow line), were chosen in Figure 2. The continuous solid yellow curve has a positive slope with ultra-high transmittance. Therefore, it can be considered as a continuous wideband mode. The SPPs, which are directly inspired by the TM beam of the incident light wave, enter the bus waveguide through $P_{1}$ and exit through $P_{2}$.

The blue line is a continuous curve with a narrow spectral width and low transmittance, and its transmission spectrum is similar to the shape of a Lorentz curve, showing a certain symmetry. Therefore, the transmission spectrum of single USRR can be considered as a discrete narrowband mode. As shown in Figure 2c, the SPPs indirectly motivated by the TM beam enter the bus waveguide from $P_{1}$ and most of them are trapped in the USRR resonant cavity. A few SPPs can return to the MIM waveguide and are propagated to $P_{2}$ to attain indirect excitation of the TM wave. The $H_{z}$ field distribution indicates that the USSR structure has a certain field intensity distributed in the whole bus waveguide.

The red line is a continuous sharp asymmetric curve with narrow spectral width and low transmittance, and its transmission spectrum is the standard Fano resonance. As shown in Figure 2a, the red curve has the lowest transmittance, which indicates the device’s ability to bind light is enhanced after the addition of double rectangular baffles to the MIM waveguide. As can be seen from Figure 2b, SPPs coupled to the USRR structure increase significantly, resulting in an increase in the field intensity of the USRR structure. The normalized $H_{z}$ field suggests that the USRR resonant cavity and the left part of the bus waveguide have a relatively strong resonance. By comparison, when the bus waveguide is examined after adding the symmetric rectangular baffles, the ability of the whole structure to gather electric field and capture SPPs is significantly strengthened, resulting in the great modulation of the asymmetric line shape of Fano resonance. 

The Fano resonance wavelength $\lambda_{1}$ can be calculated by:

(5) $$\lambda_{1}=2{\mathrm{Ln}}_{\mathrm{eff}}/J-\left(\varphi_{ref}/\pi \right)$$

where the effective resonant length of ring cavity is expressed as L; $n_{\mathrm{eff}}$ is the effective refractive index; the phase transition reflected by SPP at the MIM interface is represented as $\varphi_{ref}$; and J is the mode order (J = 1, 2, 3 …). According to Equation (5) and the analysis of the causes of Fano resonance, the parameters of the structure designed here will affect the line shape and the wavelength, thus affecting the performance of the refractive index sensor. The following changes will be made to the resonator and MIM waveguide parameters to analyze their influence on the sensitivity and figure of merit of the refractive index sensor, so as to achieve the best performance.

**Figure 3:**
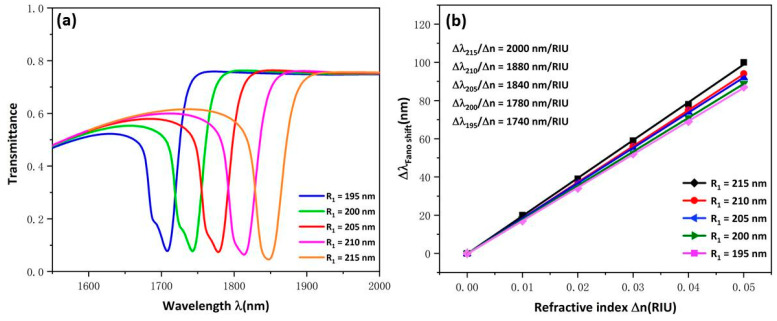
(**a**) Transmission spectra for USRR under various $R_{1}$ values; (**b**) Fitting lines of sensitivity at disparate values of $R_{1}$.

Based on the above analysis of the causes of Fano resonance, we know that the linear shape of Fano resonance can be changed by varying the broadband mode and narrow band mode, thus improving the performance of the sensor. By changing the parameters of USRR structure that generates narrowband mode, the influence of narrowband mode on the transmission performance of the whole structure was evaluated. The influence of USRR $R_{1}$ was further studied by setting $R_{1}$ to 215, 210, 205, 200 and 195 nm. In Figure 3a, as $R_{1}$ increases, the curves appear to undergo an obvious red shift with the transmittance decreased slightly. The reason for this is that due to the increase in the effective length of the USRR structure, the ability of the USRR to gather electric field increases, and eventually the resonant wavelength moves to a larger wavelength. Figure 3b shows the sensitivity fitting curve with a good linear relationship. Importantly, the change in sensitivity increases obviously from 1740 to 2000 nm/RIU as $R_{1}$ varies at 5 nm intervals. This indicates that $R_{1}$ is an important parameter to improve the sensitivity of the sensor. In practical application, proper parameters can be chosen according to the requirements of sensor manufacturing.

**Figure 4:**
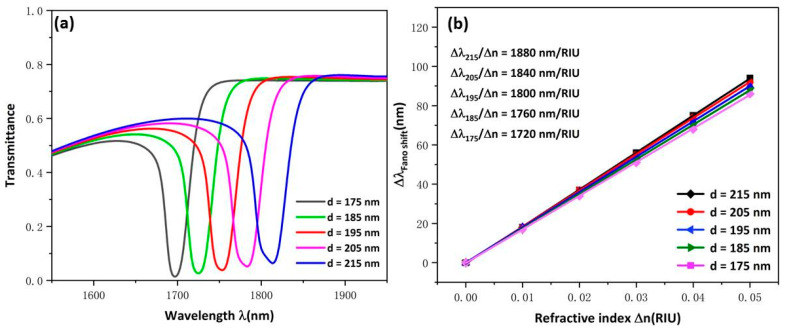
(**a**) Transmission spectra for USRR under various d values; (**b**) Fitting lines of sensitivity at disparate values of d.

Subsequently, the influence of the changed d of USRR on the whole structure’s transmission characteristics was evaluated, which were found to have increased from 175 to 215 nm. As Figure 4a shows, the transmission spectra showed an obvious redshift with d increased. The reason for this is also due to the increase in the effective length of the resonator. That the transmittance increases slightly indicates that the ability of USRR structure to gather electric field decreases slightly with the increase in cavity height. As Figure 4b shows, the sensitivity fitting curve has a good linear relationship and the sensitivity changes from 1720 to 1880. This indicates that the height of USRR structure has less influence on sensitivity than the radius; thus, changing d can improve sensitivity in a certain range.

**Figure 5:**
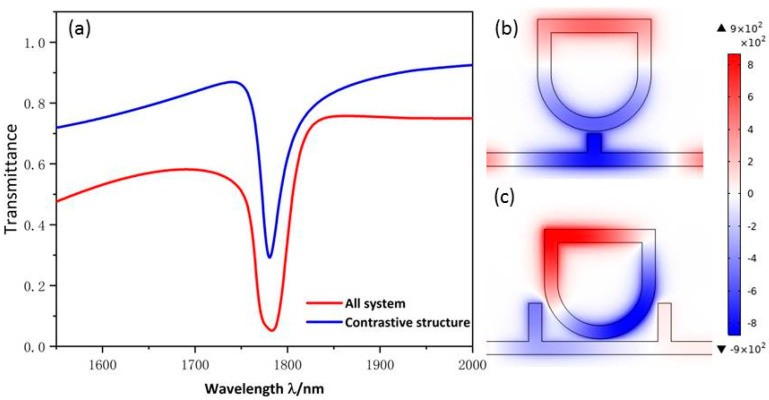
(**a**) Transmission spectra of two structures; (**b**) $H_{z}$ field distributions of the contrastive structure; (**c**) $H_{z}$ field distributions of the system.

Next, we investigated the broadband mode and evaluated the effect of broadband mode on the transmission performance of the whole structure by varying the parameters of the MIM waveguide structure that generates broadband mode. The MIM waveguide with two rectangular baffles structure and the MIM waveguide with single rectangular baffle structures were compared to explore the influence of different types of MIM waveguide on the transmission spectrum of the sensor. Both structures have the same parameters, which are set as: $R_{1}=210\;\mathrm{nm},{\;r}_{1}=R_{1}-50,\;\;d=205\;\mathrm{nm},\;\;l=440\;\mathrm{nm},\;\;g=10\;\mathrm{nm},\;\;h_{1}=145\;\mathrm{nm}\;\left(\mathrm{two}\;\mathrm{baffles}\right),\;\;\;h_{2}=70\;\mathrm{nm}\;\left(\sin\mathrm{gle}\;\mathrm{baffle}\right)$. The MIM waveguide with a single rectangular baffle structure is taken as the comparison structure. The contrast transmission spectrums of the two structures are shown in Figure 5a. The transmission spectrums show that the two structures will produce sharp curves of asymmetric Fano resonance and the wavelength remains almost unchanged. This indicates that the broadband mode has little effect on the refractive index of the sensor. It can be clearly seen that the blue curve has a high transmittance and narrow FWHM, which indicates that the ability of the whole structure to gather electric field and capture SPPs has been distinctly weakened. This can be interpreted as showing that the coupling ability of the double rectangular baffle structure to the USRR is much greater than that of the single rectangular baffle structure. As shown in Figure 5b,c, the normalized $H_{z}$ field distribution shows that when the MIM waveguide contains two rectangular baffles, the USRR structure will gather a stronger electric field and exist to a stronger resonance. Thus the following studies of waveguide structure parameters are based on an MIM waveguide with double symmetrical baffle structures.

**Figure 6:**
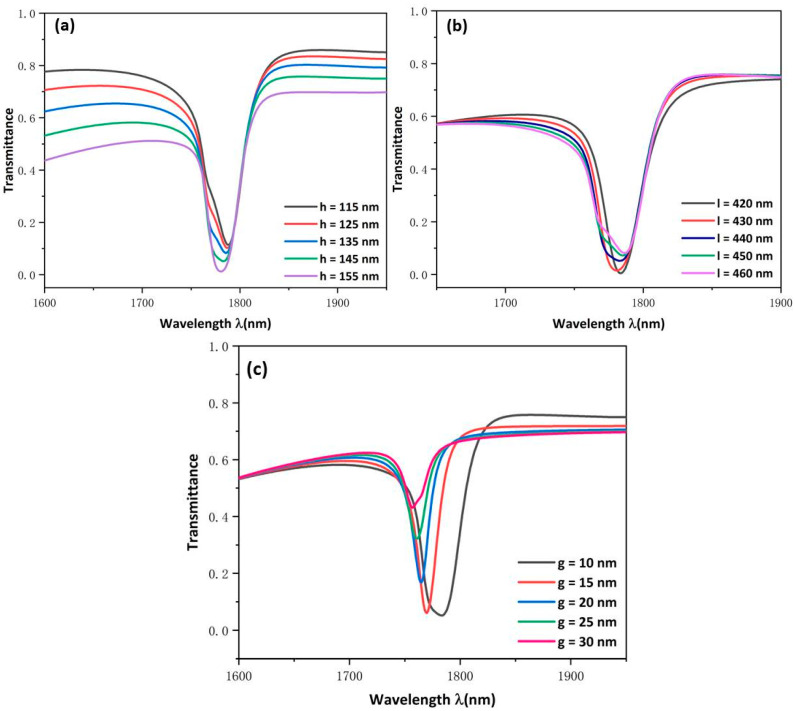
Transmission spectra at (**a**) disparate heights of rectangular baffles; (**b**) disparate distances between the two rectangular baffles; (**c**) various coupling gaps.

The influence of MIM waveguide structural parameters on system characteristics were further researched; we changed the height of rectangular baffle h from 115 to 155 nm and increased the distance between the two rectangular baffles l from 420 to 460 nm with an increase of 10 nm. As shown in Figure 6a,b, the dip position remains constant no matter how h and l were changed, which indicates that the wavelength is basically unchanged and the two parameters are not sensitive to the refractive index sensor. However, it can be seen from Figure 6a that changing h will change the shape of the curves more obviously, which transformed an almost symmetrical shape into an entirely asymmetrical shape. When h increases, the transmittance will gradually decrease, which shows that as the rectangular baffles get closer to USRR, more electric fields can be accumulated on the USRR, which ensures the good performance of the sensor. The same phenomenon is also shown in Figure 6b. When l decreased, the transmittance will gradually increase, which shows that USRR has a relatively strong resonance. Thus, the closer the rectangular baffles are to the resonator, the stronger the intensity of the field that will be gathered in the resonator and the performance of the device will be improved. The h is a more vital parameter than the distance between the two rectangular baffles in affecting the shape of the continuous broadband state. The above research indicates that transforming the h parameter can change the line’s shape without affecting its dip wavelength.

Finally, the influence of the coupling gap was evaluated by changing the g from 10 to 30 nm. Figure 6c indicates that the transmission spectrum has an obvious blue shift. The FWHM becomes narrower and the transmittance apparently increases. This indicates that increasing the coupling gap weakens the coupling of SPPs to USRR and the energy constraint of the cavity. This shows that the coupling gap g determines the low transmittance of the transmission spectrum. By balancing transmittance and FWHM, we chose 10 nm as the most optimal coupling distance for this sensor. 

**Figure 7:**
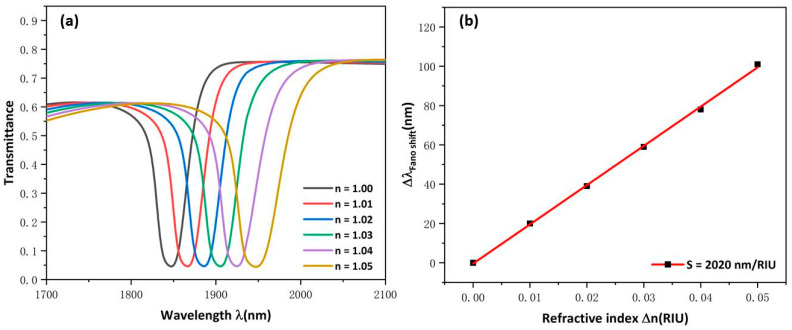
(**a**) Transmission spectra at various refractive indexes; (**b**) Fitting lines of sensitivity at disparate values of refractive indexes.

For optimal sensing performance, set the parameters to $R_{1}$ = 215 nm, d = 215 nm, h = 145 nm, l = 440 nm, g = 10 nm. The measuring theory of the refractive index sensing is that the resonance dip will change with the varying of the refractive index of surrounding materials. The refractive-index was set to 1.00, 1.01, 1.02, 1.03, 1.04, and 1.05. The transmission spectrum exhibited an obvious red shift is shown in Figure 7a. As Figure 7b shows, a maximum sensitivity and FOM of this structure were achieved, which are 2020 nm/RIU and 53.16. This is the optimal performance of the proposed structure, which is better than those which have been mentioned in the first part and are shown in Table 1. A comparison of the sensitivity of the structure indicates that the proposed structure provides better sensitivity to RI variation.

## 4. Conclusions

In this study, a novel refractive index sensor structure was designed consisting of a metal-insulator-metal waveguide with two rectangular baffles, coupled with a U-shaped ring resonator. The results show that Fano resonance, which results from the interaction of the successive wide-band mode and the discrete narrow-band mode, plays a significant role in the performance of the refractive index sensors. It was also found that the broadband mode mainly affects the line shape and FWHM of Fano resonance, and the narrowband mode mainly affects the sensitivity of sensor. A maximum sensitivity and FOM of this structure were achieved, which are 2020 nm/RIU and 53.16. The presented structure has the potential to be useful in nanophotonic sensing applications.

## Figures and Tables

**Table 1:** Comparisons of the results with recent research.

Reference	Structure Type	Sensitivity (nm/RIU)
[18]	Two baffle resonators	1650
[19]	Ring resonator	1268
[20]	D-shaped cavity	1510
[21]	Tooth cavity-coupled ring splitting cavity	1200
This work	USRR structure	2020
